# Improving plan quality and consistency by standardization of dose constraints in prostate cancer patients treated with CyberKnife

**DOI:** 10.1120/jacmp.v14i5.4333

**Published:** 2013-09-06

**Authors:** Martina Descovich, Mauro Carrara, Sara Morlino, Dilini S. Pinnaduwage, Daniel Saltiel, Jean Pouliot, Marc B. Nash, Emanuele Pignoli, Riccardo Valdagni, Mack Roach III, Alexander R. Gottschalk

**Affiliations:** ^1^ Department of Radiation Oncology University of California San Francisco San Francisco CA USA; ^2^ Medical Physics Unit, Fondazione IRCCS Istituto Nazionale dei Tumori Milano Italy; ^3^ Radiotherapy 2 Unit and Prostate Cancer Program Fondazione IRCCS Istituto Nazionale dei Tumori Milano Italy

**Keywords:** prostate, stereotactic body radiation therapy, plan optimization, dose constraints

## Abstract

Treatment plans for prostate cancer patients undergoing stereotactic body radiation therapy (SBRT) are often challenging due to the proximity of organs at risk. Today, there are no objective criteria to determine whether an optimal treatment plan has been achieved, and physicians rely on their personal experience to evaluate the plan's quality. In this study, we propose a method for determining rectal and bladder dose constraints achievable for a given patient's anatomy. We expect that this method will improve the overall plan quality and consistency, and facilitate comparison of clinical outcomes across different institutions. The 3D proximity of the organs at risk to the target is quantified by means of the expansion‐intersection volume (EIV), which is defined as the intersection volume between the target and the organ at risk expanded by 5 mm. We determine a relationship between EIV and relevant dosimetric parameters, such as the volume of bladder and rectum receiving 75% of the prescription dose (V75%). This relationship can be used to establish institution‐specific criteria to guide the treatment planning and evaluation process.

A database of 25 prostate patients treated with CyberKnife SBRT is used to validate this approach. There is a linear correlation between EIV and V75% of bladder and rectum, confirming that the dose delivered to rectum and bladder increases with increasing extension and proximity of these organs to the target. This information can be used during the planning stage to facilitate the plan optimization process, and to standardize plan quality and consistency. We have developed a method for determining customized dose constraints for prostate patients treated with robotic SBRT. Although the results are technology‐specific and based on the experience of a single institution, we expect that the application of this method by other institutions will result in improved standardization of clinical practice.

PACS numbers: 87.55.‐x, 87.55.D‐, 87.55.de, 87.55.dk

## I. INTRODUCTION

The optimization and evaluation of a radiation therapy treatment plan is a complex process involving multiple steps. As a first step, the physician delineates target and organs at risk on the planning CT scan, often with the aid of other imaging sources (PET, MRI). Then the physicist or dosimetrist runs the computer‐based optimization engine until a solution is found that meets the dosimetric parameters requested by the physician. The solution of the planning problem is particularly challenging when optimizing plans for prostate cancer patients treated with CyberKnife stereotactic body radiation therapy (SBRT). Multiple conflicting goals exist in this case, such as target coverage, conformity and homogeneity of the dose distribution, sparing of the organs at risks (bladder, rectum and urethra), and treatment time. Schlaefer et al.^(1)^ describe a powerful multicriteria optimization approach for robotic radiosurgery. This method maps clinical goals into optimization steps and enables an optimal trade‐off among different objectives by producing Pareto‐efficient solutions.

As a next step physicist, dosimetrist, and physician visually examine the dose distribution on the axial, sagittal, and coronal plane of the CT scan, and evaluate the target coverage and dose received by organs at risk using dose‐volume histogram (DVH) analysis. A plan is deemed clinically acceptable if tumor coverage and dose tolerance to critical structures are within standard limits and/or meet physician expectations. The use of standard dose criteria in evaluating plan quality has however some limitations.® In particular, standard dose criteria do not account for the mutual disposition of target and organs at risk, which determines the difficulty of each individual treatment. In addition, modern radiotherapy equipment might provide superior dose distribution with lower dose to organs at risk, compared to conventional treatment modalities.

The main drawback of using equal dose criteria for every patient is that suboptimal treatment plans might still meet those criteria and, therefore, be deemed acceptable. Although physicist and physician experiences play a critical role in pushing the plan quality to the optimal limit, the process is somewhat subjective and lacks quantitative guidelines. Consequently, plans deemed acceptable might be far from the unknown optimality.

The goal of this study is to help establish an objective method to evaluate the plan's quality, which accounts for the individual patient's geometry and is independent of physician and planner experiences. Prior to starting the optimization process, the method provides dose objectives for the individual case. These objectives are derived from a database of patients with similar characteristics and, therefore, can be considered achievable.

Differences in target and critical structures definition among physicians introduce a source of uncertainty in establishing a user independent quality control method based on geometric criteria. Previous studies reported significant differences in treatment planning and DVH analysis due to uncertainty in target^(3)^ and critical structures contouring.^(4)^

In this work, we focus on prostate patients planned and treated with CyberKnife SBRT. However, a similar methodology can be developed for other treatment sites or treatment modalities.

The a priori knowledge of achievable dose constraints facilitates the treatment planning process and results in better plan quality. In addition, comparing dosimetric parameters of a newly developed treatment plan with institution‐specific statistics provides a tool to control the plan quality and to improve the consistency among similar plans. We expect that this method will also facilitate interinstitution comparison of plan quality and eventually lead to standardization of clinical practice.

## II. MATERIALS AND METHODS

The proposed method was developed for prostate patients treated with SBRT on the CyberKnife system (Accuray Inc., Sunnyvale, CA). In SBRT treatments, a high biologic‐equivalent dose (BED) is delivered in few fractions (typically 2–5) with highly conformal distribution around the prostate and steep dose gradient at its periphery. Small treatment margins are possible, as the position of the prostate is monitored during the course of treatment every 30–60 seconds. Xie et al.^(5)^ analyzed the intra‐fraction prostate motion in 21 patients (105 fractions) treated with the CyberKnife system. They concluded that with an imaging interval of 30 seconds, target margins could be kept as low as 1 mm and that 2 mm margin would be required if the imaging interval was increased to 60 seconds. Gottschalk et al.^(6)^ reported similar results.

Several SBRT treatment protocols are reported in the literature for the treatment of the prostate as both mono‐therapy and post‐pelvic irradiation boost.^7^, ^8^, ^9^, ^10^ The dose regimens and constraints for the organs at risk have been mainly derived from the experience in HDR brachytherapy, a well‐established modality for delivering fewer high‐dose fractions as monotherapy^(11)^ or in combination with whole pelvis radiation therapy.^(12)^ Table 1 reports a comparison of dose scheme and constraints published in the literature for HDR^(13)^ and robotic SBRT protocols.^7^, ^8^, ^9^, ^10^

In order to establish quality control criteria for treatment plan evaluation at our institution, we reviewed the dosimetric parameters obtained on a database of 25 prostate patients. For consistency in plan quality, we considered only plans generated with the Iris variable aperture collimator (Accuray Inc.), which enables to deliver multiple‐size radiation fields at each robot position.^(14)^ Iris treatments do not require physical collimator exchange and are delivered in a single path traversal. Previous studies have shown that plans generated with three collimator apertures deliver better treatment quality (improved dose conformity, homogeneity and/or coverage, and lower number of monitor units) compared to a single circular collimator.^(^ ^14^ ^,^ ^15^ ^)^ The Iris collimator, therefore, offers the benefits of multiple‐field‐size treatments without increasing planning and delivery time.

The use of existing clinical plans of comparable treatment characteristics enabled us to determine dosimetric criteria that are achievable for a given patient anatomy.

**Figure 1:**
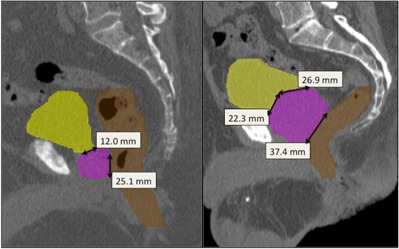
Anatomy of prostate patients. Examples of favorable (left) and unfavorable (right) patient anatomy for a prostate SBRT treatment. Linear dimensions of bladder‐PTV and rectum‐PTV interface are displayed on the same scale.

The geometric distribution of organs at risk relative to the target plays a critical role in determining the achievable dose constraints. For example, standard dose criteria can be easily achieved for a patient with a small prostate and a large separation between target and organs at risk (Fig. 1, left). On the other hand, it is very difficult to achieve the same dose constraints in a patient with a large prostate and significant interface between target and organs at risk (Fig. 1, right). Treatment preparation, such as rectum and bladder filling, and the use of rectal balloons (for prostate immobilization and rectal wall sparing), bladder catheters (for constant bladder filling), and Foley catheters (for urethral delineation) influence the patient geometry and organ disposition. At our institution, patients are simulated with empty rectum and bladder (due to the prolonged treatment time, it is not feasible for the patients to maintain full bladder). In addition, the use of endorectal coils during MRI acquisition, and Foley catheters during simulation or treatment is not allowed to prevent deformation in the prostate gland.

In order to quantify the geometric relationship in the 3D space between the target volume and the organs at risk we used the concept of expansion‐intersection histogram proposed by Tomatis et al.^(16)^ We define the intersection volume between the target and the organ at risk expanded by 5 mm as EIV, expansion‐intersection volume. A large EIV indicates that a large area of organ at risk faces the target, leading to a more difficult plan optimization.

**Table 1:** Literature‐based treatment parameters for HDR and robotic SBRT protocols

*Parameter*	*HDR* ^(13)^	*San Diego* ^(8)^	*UCSF* ^(9)^	*Erasmus* ^(7)^	*UCLA* ^(10)^
Total dose	19 Gy	38 Gy	19 or 38 Gy	38 Gy	36.25 Gy
Fractions	2	4	2 or 4	4	5
Prescription		>50%	>60%	>67%	88%‐92%
PTV margin	none	2–5 mm/0 post.	2 mm/0 post.	3 mm/0 post.	5 mm/3 mm post.
PTV	${V100\%}^{a}\;\geq 90\%$	${V100\%}^{a}\;\geq 95\%$	${V100\%}^{a}\;\geq 95\%$	${V100\%}^{a}\;\geq 95\%$	${V100\%}^{a}\;\geq 95\%$
Rectum	${V75\%}^{a}\;<1\;\text{cc}$	Wall $V{100\%}^{a}\;=\;0\;{\text{Mucosa}}^{b}\;{V75\%}^{a}\;=\;0$	${V75\%}^{a}\;<2\;\text{cc}$	${\text{Wall}\;V100\%}^{a}\;=\;0$
${\text{Mucosa}}^{b}\;{V75\%}^{a}\;=\;0\;{V85\%}^{a}\;<1\;\text{cc}$	${V50\%}^{a}\;<50\%\;{V80\%}^{a}\;<20\%\;{V90\%}^{a}\;<10\%\;{V100\%}^{a}\;<5\%$
Bladder	${V75\%}^{a}\;<1\;\text{cc}$	${V120\%}^{a}\;=\;0$	${V75\%}^{a}\;<3\;\text{cc}$	${V110\%}^{a}\;=\;0\;{V100\%}^{a}\;<1\;\text{cc}$	${V50\%}^{a}\;<40\%\;{V100\%}^{a}\;<10\%$
Urethra ${V125\%}^{a}\;<1\;\text{cc}\;{V120\%}^{a}\;=\;0\;{V120\%}^{a}\;<10\%\;{V120\%}^{a}\;=\;0$

a Vxx: Volume of structure (PTV or organ at risk) receiving xx% of prescription dose.

b Mucosa: solid structure formed by a 3 mm contraction of the rectal wall.

### A. Treatment planning technique

For each patient, three gold fiducial markers are implanted into the prostate gland at least one week prior to CT simulation. The gold markers provide a reference frame to localize the position of the prostate in the X‐ray images acquired during treatment. A CT scan using 1.5 mm slice thickness is acquired, together with T1‐ and T2‐weighted MRI imaging sequences. The CT and T2‐weighted MRI scans are coregistered using a point‐based registration to match the fiducial location. While the T2 sequence provides optimal visibility of prostate and urethra, the T1 sequence helps identify the fiducial markers, which produce a low signal in the T2‐weighed images. The planning target volume (PTV) is created by a uniform 2 mm expansion of the prostate (contoured on the T2 sequence), except posteriorly to protect the rectum. The physician also contours organs at risk such as bladder, rectum, urethra, penis, testicles, bowel, and femoral heads. All organs at risk, except the urethra, are contoured on the planning CT. The bladder is contoured from its base to the dome. The rectum is contoured from the anus to the recto‐sigmoid flexure.

The “sequential optimization” (or “stepwise optimization”) method is used for plan generation.^(1)^ This method provides a flexible approach to solve the planning problem and optimize multiple conflicting goals reproducing the decision‐making process of a clinician. Unlike inverse planning algorithms, which use relative weights and a single mathematical function to optimize all planning goals at once, sequential optimization addresses each goal separately in a predefined order. After each step, the feasibility of the solution is retained or relaxed by a user‐defined value to improve plan quality at the next step. However, in the CyberKnife implementation of the sequential method, the relaxation parameter applies only to the last step in the sequential chain. This prevents major improvements in plan quality after step 2. The sequential optimization method possesses the right characteristics for scripting. Scripts (or plan templates) greatly reduce the variability of different treatment planning skills and help improve the standardization among similar plans.

For prostate patients, a planning template is used at our institution. Beams intersecting the testicles are disabled, and field sizes ranging from the largest aperture that fits the PTV con‐tour down to the 12.5 mm diameter opening are selected. Three asymmetric shells are created around the PTV with larger margins in the lateral direction and tighter margins in the anterior‐posterior direction, to improve rectum and bladder sparing. Shells are tuning structures used by the optimization algorithm to achieve a conformal dose distribution around the target and control the dose gradient at its periphery. The distance between shell and PTV is 1–4 mm for the first shell, 6–12 mm for the second shell, and 15–25 mm for the third shell. The use of thin shells and dose‐volume constraints for planning robotic radiosurgery is reported in the paper by Schlaefer et al.^(17)^ This article discusses a specific example for a prostate case.

A maximum of 200 monitor units (MUs) per beam per fraction is set in order to limit the beam‐on time from each direction, therefore preventing a “finger‐like” dose distribution. The dose distribution has “fingers” when the isodose line corresponding to 50% of the prescription dose is not shaped around the target, but protrudes for several centimeters away from it. Properly placed shells also help reducing this effect. The maximum MU per node is set to be slightly larger than the maximum MU per beam to enable the use of multiple beams per node, while avoiding skin hotspots due to predominant beam entry points. In some cases, the definition of a planning‐specific target that does not overlap with bladder and rectum, and does not include the urethra, facilitates the planning process.

The sequential optimization script is run to achieve the following clinical goals: i) to cover 95% of PTV with 100% of the prescription dose; ii) to keep the volume of urethra receiving 120% of the prescription dose (V120%) less than 0.1 cc; iii) to minimize the volume of rectum receiving 75% of the prescription dose (V75%); iv) to minimize the volume of bladder receiving 75% of the prescription dose (V75%); and v) to obtain a dose distribution highly conformal around the target, with no hot spots in normal tissue.

After a plan of acceptable quality is achieved, beam and time reduction tools are used to obtain the best compromise between treatment efficiency and dosimetric quality.^(18)^

### B. Training cohort

Eleven of the 25 patients in the training cohort were treated with SBRT monotherapy using a dose of 38 Gy in 4 fractions. Fourteen patients were treated with SBRT boost. Among these, seven patients received a dose of 19 Gy in 2 fractions and seven patients received a dose of 21 Gy in 2 fractions, according to a dose escalation protocol. To assess the robustness of the method to contouring uncertainties, patients contoured by three physicians were included in our study. Two physicists were involved in the treatment planning process.

The average PTV volume is $58.3\;{\text{cm}}^{3}$ (range $11.3‐148.3\;{\text{cm}}^{3}$), the average bladder volume is $130.8\;{\text{cm}}^{3}$ (range $47.8‐385.5\;{\text{cm}}^{3}$), and the average rectum volume is $102.0\;{\text{cm}}^{3}$ (range $45.4‐365.8\;{\text{cm}}^{3}$). For all plans, target coverage ≥ 95%, and prescription isodose‐line > 60% are achieved. The average number of beams is 209 (range 112–360), and the average treatment time (excluding setup time) is 43 minutes (range 27–60 minutes).

For each plan, the following parameters are recorded and analyzed: i) EIV of rectum and bladder; ii) V75% of rectum and bladder; iii) V120% of urethra; iv) conformity index (nCI).

The conformity index describes how well the prescription isodose volume (PIV) conforms to the target volume (TV) and it is defined as: $\text{nCI}\;=\;\text{TV}\;\times \;\text{PIV}\;/\;({\text{TIV})}^{2}$, where TIV is the target volume inside the prescription isodose volume. The average nCI was 1.24 (range 1.14–1.41), and the average urethra V120 was 0.03 cc (range 0–0.19 cc).

In this analysis, we use V75% of bladder and rectum and V120% of urethra as the key dosimetric parameters to evaluate plan quality. This choice reflects our clinical practice and is based on the experience with HDR brachytherapy.^(12)^

The V75% values of bladder and rectum are plotted as a function of the EIV values of bladder and rectum. A linear regression analysis is performed in order to model the relationship between these two parameters. Prediction intervals, representing the intervals where 90% and 95% of future observations will fall, are also calculated. The prediction intervals enable to compare a new plan to our institutional standards and provide a method for quality control.

### C. Validation cohort

The robustness of the method is tested on five new prostate cases. For all cases, the EIVs of bladder and rectum are calculated a priori and used to determine the expected values of V75%. These values serve as guidelines during the plan optimization process.

## III. RESULTS

### A. Training cohort

A linear correlation between V75% and EIV is found for EIV values up to $10\;{\text{cm}}^{3}$, followed by a saturation of V75% for larger EIV values.

**Figure 2:**
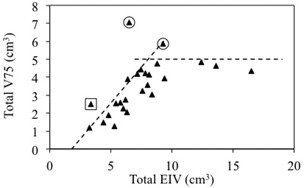
Correlation between V75% and EIV of bladder and rectum. V75% of bladder plus rectum (total V75%) plotted as a function of EIV of bladder plus rectum (total EIV). The open circles represent data points that are above the dose constraints: total $V75\%\;<5\;{\text{cm}}^{3}$. The open square represents the data point that is above the empirical threshold line.

Figure 2 reports the V75% of bladder plus rectum (total V75%) plotted as a function of the EIV of bladder plus rectum (total EIV). For all but two plans (marked with an open circle) our institution's dose constraints are achieved. In addition, for patients with small EIV, a total V75% considerably lower than the limit is obtained. From this plot, it emerges that all data points should fall below two lines: a first threshold line representing the institution‐specific dose constraints (i.e., total $V75\%\;<\;5\;{\text{cm}}^{3}$), and a second, empirical threshold line representing a feasible improvement in dosimetric parameters due to favorable patient anatomy (i.e., small EIV). One plan (marked with an open square in Fig. 2) fulfills the total V75% dose constraints (line 1), but is over the previously defined empirical threshold line (line 2).

The threshold lines were used to predict achievable V75% values of bladder plus rectum for the three outliers, and the treatments were replanned. In all three cases, dose objectives below the threshold could be achieved for bladder and rectum, while maintaining 95% PTV coverage, good dose conformity (nCI 1.17–1.24) and sparing of urethra (V120% < 0.1cc). An experienced radiation oncologist reviewed the new plans and compared them with the original versions. In all three cases, an improvement in plan quality was noted.

**Figure 3:**
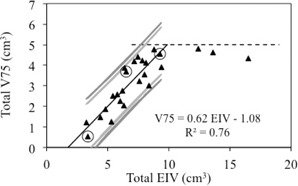
Correlation between V75% and EIV of bladder and rectum — re‐optimized plans. V75% of bladder plus rectum (total V75%) plotted as a function of EIV of bladder plus rectum (total EIV). Correlation line and prediction intervals are displayed. The open circles represent data for the re‐optimized treatment plans.

Figure 3 is an update of Fig. 2, where the data for the three outliers are replaced with the replanned data (marked as open circles). A regression analysis for the data points with $\text{EIV}<\;10\;{\text{cm}}^{3}$ provides a reasonable linear correlation $(R^{2}\;=\;0.76)$ between the total V75% and EIV. The 90% and 95% prediction intervals are also displayed. For data points with $\text{EIV}\;>\;10\;{\text{cm}}^{3}$, the horizontal threshold line representing the institution‐specific dose constraints (total $V75\%\;<\;5\;{\text{cm}}^{3}$) provides the method for quality control, and the prediction intervals are not calculated.

### B. Validation cohort

**Figure 4:**
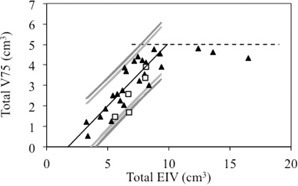
Correlation between V75% and EIV of bladder and rectum — validation data. V75% of bladder plus rectum (total V75%) plotted as a function of EIV of bladder plus rectum (total EIV). The open squares represent the validation data.

For all new prostate cases, it was possible to obtain clinically acceptable plans with (total EIV, total V75%) coordinates falling within the prediction intervals. Figure 4 displays the location of the new data points (open squares) in the (total EIV, total V75%) graph. The a priori knowledge of achievable V75% values facilitates the treatment planning process. Plans were generated by a physicist specialized in robotic radiosurgery planning in less than one day.

## IV. DISCUSSION

We presented an objective method, based on clinical experience, to evaluate the quality and consistency of prostate SBRT plans. This method enables to determine whether a satisfactory plan has been obtained without relying on personal experience. In our definition, a satisfactory plan meets the clinical goals in terms of target coverage, organs at risk sparing, and dose conformity, and therefore is consistent with the clinical objectives at our institution.

The linear correlation between EIV and V75% of bladder and rectum confirms that the dose delivered to bladder and rectum increases with increasing extension of the organs area facing the target. For a given EIV value, the linear fit enables to determine a priori optimal dose objectives for the organs at risk. This knowledge facilitates the treatment planning process in terms of both planning efficiency and plan quality, and provides a means to detect abnormal plan behavior.

**Figure 5:**
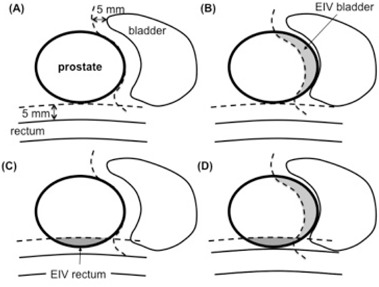
Geometrical disposition of prostate and organs at risk.

In our analysis, the dose objectives for bladder and rectum are combined in a single parameter, the total V75%. The correlation between the total V75% and total EIV is indeed stronger than the correlation between V75% and EIV of bladder and rectum analyzed separately. This behavior can be explained by considering possible geometrical mutual dispositions of prostate and organs at risk, as displayed in Fig. 5. In the schematic drawings (Fig. 5(a) to 5(d)), prostate, bladder and rectum have the same size, but different mutual disposition. In Fig. 5(a), bladder and rectum are far from the prostate and therefore their EIVs are small; in (b) bladder EIV is large and rectum EIV is small; in (c) bladder EIV is small and rectum EIV is large; in (d) both organs are facing the prostate and therefore their EIVs are large. In Figs. 5(b) and (c), the treatment plan can be optimized so that the 5% fraction of PTV volume receiving less than 100% of the prescribed dose (acceptable PTV coverage is 95%) is entirely concentrated at the interface with the bladder or with the rectum, respectively. In this way, the portion of bladder or rectum facing the prostate is mostly spared because the dose gradient starts already inside the prostate. Conversely, in Fig. 5(d) both organs at risk are facing the target, and a compromise for the location of the dose gradient has to be reached. Figure 5 demonstrates that the dose delivered to the rectum depends not only on its proximity to the target, but also on the proximity of the bladder to the target. Similarly, the dose delivered to the bladder depends on the rectum location.

In the optimization algorithm, user‐defined dose constraints are set in order to minimize the dose delivered to bladder and rectum. By properly adjusting constraints and optimization steps in the sequential workflow, it is possible to either balance the V75% of bladder and rectum to a similar value, or to prioritize the sparing of one organ versus the other, depending on the detailed clinical assessment.

The total EIV analysis returns the value of V75% of bladder plus rectum for a given patient anatomy. The achievable V75% of rectum and bladder, separately, can be estimated as follows:

(1) Bladder V75%=f*Total V75%

(2) Rectum V75%=(1−f)*Total V75%

where *f* is a parameter with values ranging between 0 and 1. In general, f = 0.5 can be used. However, for large EIV it is generally preferred to keep rectum $V75\%\;<\;2\;{\text{cm}}^{3}$ and bladder $V75\%\;<\;3\;{\text{cm}}^{3}$. In this case, f = 0.6 is used.

**Figure 6:**
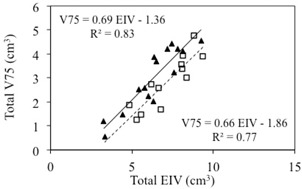
Correlation between V75% and EIV of bladder and rectum — conformity index. Total V75% as a function of total EIV for plans with low nCI (black triangles) and high nCI (open squares). The two correlation lines are also displayed.

A similar interplay is observed between the sparing of the organs at risk and the dose conformity around the target. Figure 6 shows the total V75% versus the total EIV separated in two groups: 1) plans with nCI < 1.25 (black triangles), and 2) plans with nCI > 1.25 (open squares). A slightly stronger linear correlation between V75% and EIV emerges from the two groups ($R^{2}=\;0.83$ and $R^{2}\;=\;0.77$ for plans with low nCI and high nCI, respectively). Two almost parallel correlation lines are obtained from the two datasets, demonstrating that for a given EIV value, there are two alternative approaches to optimize the V75% values, one corresponding to a more conformal plan (nCI < 1.25) and one corresponding to a less conformal plan (nCI ≥ 1.25). Although some differences are observed between the linear fits for the different conformity levels, these results are not statistically significant. Prioritizing dose conformity, sparing of bladder and sparing of rectum is a clinical decision that needs to be evaluated on a case‐by‐case basis. For patients with favorable anatomy, physicians may also consider escalating the dose, while maintaining safe dose limits to the organs at risks. A method for estimating the limits of dose escalation for prostate radiotherapy has been described by Roach et al.^(19)^

We have established a method to determine achievable dose constraints for bladder and rectum prior to treatment planning. This method is based on an empirical relationship between EIV and V75% observed on a cohort of 25 patients. The choice of investigating the 75% dose level reflects our clinical practice and it is derived from the well‐established experience in HDR brachytherapy. Choosing a different dose level might lead to a different relationship with EIV. A detailed characterization of the sensitivity to the selected dose level is outside the scope of this work.

The focus of this work is in the proposed methodology rather than the specific criteria and results obtained. The proposed criteria should not be considered as dose tolerance for critical organs, but as a guideline to improve institutional standards. Indeed, one limitation of this method is that it is institution‐ and technology‐specific. However, being based on a measurable quantity (such as the EIV), this method could be easily validated by other institutions for intercomparison of plan quality. Differences in target and critical structures definition among physicians/institutions result in different dose‐volume histogram (DVH) characteristics and make it difficult to compare clinical outcomes. Therefore, it is important to evaluate dosimetric parameters in relationship to a parameter accounting for the proximity of the organs at risk to the target. Interinstitution studies are required to further test the robustness of this method to contouring uncertainties.

We believe that this method could be particularly useful to institutions starting a new prostate SBRT program, or exploring advantages of new technologies.

## V. CONCLUSIONS

A new method for objectively evaluating the quality of prostate SBRT plans is proposed. This method provides achievable dose constraints for bladder and rectum taking into account the patient's anatomy. The knowledge of achievable dose constraints facilitates the treatment planning process, identifies suboptimal plans, and provides a means to control plan quality and consistency. This method is now routinely used in our clinical practice.

## ACKNOWLEDGMENTS

The authors would like to thank Fondazione ProADAMO for its support to the study and Tiziana Rancati for her precious comments to the manuscript.
